# Active transportation is associated with lower obesity risk: generalized structural equations model applied to physical activity

**DOI:** 10.1590/0102-311XEN035624

**Published:** 2025-01-13

**Authors:** Fabian Leonardo Muñoz, Sonia Alejandra Pou, Humberto Llinas Solano, María del Pilar Diaz

**Affiliations:** 1 Instituto de Investigaciones en Ciencias de la Salud, Universidad Nacional de Córdoba, Córdoba, Argentina.; 2 Escuela de Nutrición, Universidad Nacional de Córdoba, Córdoba, Argentina.; 3 Universidad del Norte, Barranquilla, Colombia.

**Keywords:** Obesity, Physical Activity, Latent Variable Modeling, Surveys, Bicycling, Obesidad, Actividad Física, Modelado de Variable Latente, Encuestas, Ciclismo, Obesidade, Atividade Física, Modelagem de Variáveis Latentes, Inquéritos, Ciclismo

## Abstract

This study aimed to identify latent (unobservable) dimensions representing specific physical activity-related behaviors and explore their potential effects on obesity burden and spatial distribution in Colombia. A cross-sectional study (n = 9,658) was conducted based on the *Colombian National Survey of Nutritional Status*. A generalized structural equations model was proposed, combining exposure and measurement models to define a disease model. Modeling identified latent dimensions of physical activity focused on screen time and means of transportation and estimated their direct and indirect effects on obesity occurrence. Mapping techniques were used to illustrate adherence to these dimensions. The latent dimensions identified were named “Screens use” and “Active transportation”; the latter was inversely associated with obesity occurrence (p = 0.004), with the use of bicycles being the dominant variable, contrasting with the use of motor vehicles. The mapping showed that departments with the highest adherence to the “Active transportation” construct have a lower prevalence of obesity. Bicycle use, as opposed to non-active transportation, represented a dimension of physical activity-related behaviors with a protective effect against obesity. This suggests that active transportation may be a crucial factor in the designing preventive interventions. Moreover, social inequalities may be contributing to the obesity epidemic and physical activity behaviors in Colombia, requiring equitable and multisectoral responses.

## Abstract (es)

Este estudio buscó identificar dimensiones latentes (no observables) que representan comportamientos específicos relacionados con la actividad física y explorar sus efectos potenciales sobre el impacto de la obesidad y en la distribución espacial en Colombia. Se realizó un estudio transversal (n = 9.658) con base en la *Encuesta Nacional de Situación Nutricional*. El modelo con ecuaciones estructurales generalizadas propuesto combinó modelos de exposición y medición para definir un modelo de enfermedad. El modelo identificó dimensiones latentes de la actividad física centrándose en el tiempo frente a la pantalla y en los medios de transporte y estimó sus efectos directos e indirectos sobre la aparición de obesidad. Se utilizaron técnicas de mapeo para ilustrar la adhesión a estas dimensiones. Las dimensiones latentes identificadas fueron denominadas “Uso de pantallas” y “Transporte activo”; esta última se asoció inversamente con la aparición de obesidad (p = 0,004), y el uso de bicicletas en detrimento del de vehículos automotores fue la variable dominante. El mapeo mostró que los departamentos con mayor adhesión al constructo “Transporte activo” presentan una menor prevalencia de obesidad. El uso de bicicletas en lugar del transporte inactivo configuró una dimensión de conductas relacionadas con la actividad física con efecto protector frente a la obesidad. Esto sugiere que el transporte activo puede ser un factor importante que tener en cuenta al planificar intervenciones preventivas. Además, las desigualdades sociales pueden estar impulsando la epidemia de obesidad y las conductas de actividad física en Colombia, lo que requiere respuestas equitativas y multisectoriales.

## Abstract (pt)

Este estudo buscou identificar dimensões latentes (não observáveis) que representam comportamentos específicos relacionados à atividade física e explorar seus efeitos potenciais no impacto da obesidade e na distribuição espacial na Colômbia. Um estudo transversal (n = 9.658) foi realizado com base na *Pesquisa Nacional da Situação Nutricional da Colômbia*. A modelagem com equações estruturais generalizadas proposta combinou modelos de exposição e medição para definir um modelo de doença. A modelagem identificou dimensões latentes da atividade física com foco no tempo de tela e nos meios de transporte e estimou seus efeitos diretos e indiretos na ocorrência de obesidade. Técnicas de mapeamento foram utilizadas para ilustrar a adesão a essas dimensões. As dimensões latentes identificadas foram denominadas “Uso de telas” e “Transporte ativo”; esta última foi inversamente associada à ocorrência de obesidade (p = 0,004), sendo o uso de bicicletas em detrimento do de veículos automotores a variável dominante. O mapeamento mostrou que os departamentos com maior adesão ao construto “Transporte ativo” apresentam menor prevalência de obesidade. O uso da bicicleta em detrimento do transporte inativo configurou uma dimensão de comportamentos relacionados à atividade física com efeito protetor contra a obesidade. Isso sugere que o transporte ativo pode ser um fator importante a ser considerado no planejamento de intervenções preventivas. Além disso, as desigualdades sociais podem estar impulsionando a epidemia de obesidade e os comportamentos de atividade física na Colômbia, o que exige respostas equitativas e multissetoriais.

## Introduction

Overweight and obesity are among the major global public health issues of the 21st century ^1^ ^,^ ^2^. Despite progress in understanding the obesity epidemic, strategies to control and reduce it, many focusing on promoting physical activity and healthy lifestyles, have not yet produced the expected results. In recent decades, as the world’s population has undergone a nutrition transition, a decline in physical activity levels has been observed ^3^. In line with this global trend, the prevalence of obesity in Colombia increased from 13.7% in 2005 to 18.7% in 2015, and leisure-time physical activity decreased significantly from 2005 to 2010 ^4^. Interestingly, Colombia holds valuable information, rigorously collected via a nationwide survey called the *Colombian National Survey of Nutritional Status* (ENSIN; *Encuesta Nacional de Situación Nutricional*), which has not yet been fully used for a detailed study of the links between physical activity patterns and obesity. This could provide new insights into this issue.

Physical activity, broadly defined as all forms of movement including during leisure time, for transport to get to and from places, and/or as part of a person’s work ^5^, is considered a modifiable factor associated with many health conditions, including obesity and its associated chronic diseases ^6^ ^,^ ^7^. Its health advantages include psychological effects, maintenance of physical fitness, and promotion of healthy behaviors, which result in greater well-being and quality of life ^8^. In particular, the favorable effects of physical activity on maintaining a healthy body weight are well established, but most of the evidence has focused on overall physical activity and regular physical activity during leisure time ^7^. Comparatively, fewer studies have focused on other domains of physical activity, such as active transportation (cycling and walking to work) or sedentary behavior (i.e., sitting or lying down activities during waking hours that require low energy expenditure). Although interest on this subject is growing, the role of different domains of physical activity on obesity occurrence needs a better explanation.

In physical activity research, some review studies have explored the impact of time spent in sedentary behavior on several health outcomes, including obesity in adulthood ^9^ ^,^ ^10^ ^,^ ^11^. Overall, it is agreed that the evidence needed to understand this complex association is inconclusive. Regarding the link between obesity/adiposity and sedentary behaviors, more consistent associations have been shown for screen time (mainly TV viewing) ^9^ and car use ^10^, although in general these factors have been studied independently. The means of transportation is another topic increasingly studied since strong evidence points that active transport behavior (primarily walking and cycling) can result in substantial health benefits ^12^ ^,^ ^13^ ^,^ ^14^ ^,^ ^15^. However, while some studies report that active transportation appears to be associated with a lower risk of obesity or healthier body weight ^16^ ^,^ ^17^, others note that the evidence is still unclear ^13^ ^,^ ^18^ ^,^ ^19^.

Given the above, it is reasonable to assume that the notion of physical activity involves a complex phenomenon that can be represented as a structure or system of interrelated variables, which presents a methodological challenge to its study. The generalized structural equation model (GSEM) emerges as a useful analytic strategy to address this challenge because it allows examining theoretical connections between variables, whether observable or not directly observable, for a comprehensive analysis. The classic models are insufficient to evaluate the effects of measurable and latent variables associated with obesity. Finding latent dimensions, GSEM enables verifying the suitability of theoretical models in the study population, thereby offering a more robust approach to understanding the intricate dynamic between multiple variables ^20^ ^,^ ^21^.

This study aimed to (a) identify latent (unobservable) dimensions representing specific physical activity-related behaviors and estimate their direct and indirect effects on obesity, and (b) explore the spatial distribution of the population scores of adherence to the identified latent variables of physical activity and the obesity prevalence in Colombia. We hypothesized that there are underlying dimensions (not directly observable) representing non unidimensional aspects of the physical activity associated with the presence of obesity.

## Methods

### Study design and sample

In this cross-sectional study, the population-based dataset corresponding to the last ENSIN was used ^22^. The last edition of this survey was conducted in 2015, following a multistage stratified random sampling design, with urban and regional coverage. Further details of the ENSIN survey can be found elsewhere ^2^.

First, a subset of 10,635 people aged 18-64 years with complete information on physical activity and energy intake was extracted from the total number of participants in the 2015 ENSIN. The exclusion criteria included pregnant or breastfeeding women, incomplete anthropometric data, report of physical or mental disabilities, and unreliable energy intake reporting (< 1st percentile or > 99th percentile). After applying the criteria, the final sample size was 9,658.

### Data and instruments

#### Sociodemographic characteristics

The following sociodemographic variables were selected from the ENSIN in order to characterize the study population and/or adjust the estimates: sex (male, female), age group in years (18-29, 30-49, 50-64), ethnicity (people of African ancestry, Indigenous people, people of other ethnic origins), schooling level (highest level of schooling attained: primary, secondary, or tertiary), and wealth index (levels by quartiles: 1 - low, 2 - middle-low, 3 - middle-high, 4 - high). In particular, the ENSIN administers a structured questionnaire on socio-demographic and economic characteristics to the household head, developed by the *Colombian Demographic and Health Survey*.

#### Anthropometric variables

In this study, obesity was defined as a body mass index (BMI) greater than or equal to 30, following the World Health Organization (WHO) criterion. The WHO states that BMI, estimated as weight (kg)/height^2^ (m^2^), is a surrogate marker of fatness ^23^ and can therefore be considered a suitable proxy for obesity.

The ENSIN used anthropometric measurements of height and weight carried out by trained personnel. Subjects were asked to be barefoot and wear light clothing. A stadiometer (1mm precision, Shor Productions LLC, https://weighandmeasure.com/shorrboards) and a digital scale (model 874, 100g precision, SECA; https://www.seca.com) were used.

#### Behavioral factors

Data on physical activity and dietary intake from the ENSIN was considered in the modeling phase of this study. Specifically, to construct the physical activity dimensions (latent construct), the study considered time (minutes/week) spent using different modes of transport (passive transport in a motor vehicle; cycling or walking) and using screens (computer, smartphone, or other digital devices for playing video games). Other behavioral variables derived from the ENSIN data were leisure time (minutes/week) dedicated to moderate or vigorous physical activity (from the results of the *International Physical Activity Questionnaire* - IPAQ), and daily energy intake (kcal/day, estimated from two nonconsecutive 24-hour recalls applied by the ENSIN) ^2^.

For physical activity, the ENSIN employs the IPAQ (long format) ^24^ and a structured questionnaire on sedentary behaviors. Dietary data was obtained from the 24-hour diet recall technique. Further details are available in previous publications ^2^.

### Statistical analysis

To describe the sociodemographic characteristics of the sampled individuals, frequency tables were constructed and summary measures were estimated. The chi-square test and the Mann-Whitney U test were used for comparative purposes.

In the modeling phase of the study, based on the available data from the ENSIN survey, two physical activity dimensions were theoretically proposed, considering their potential impacts on the energy balance: (i) sedentary behaviors mediated by screens (leisure time that the person spends on computer and smartphone or playing video games, measured in minutes/week), and (ii) the modes of transport in daily life (time spent on motor vehicle, cycling, and walking as means of transport, in minutes/week).

**Figure 1:**
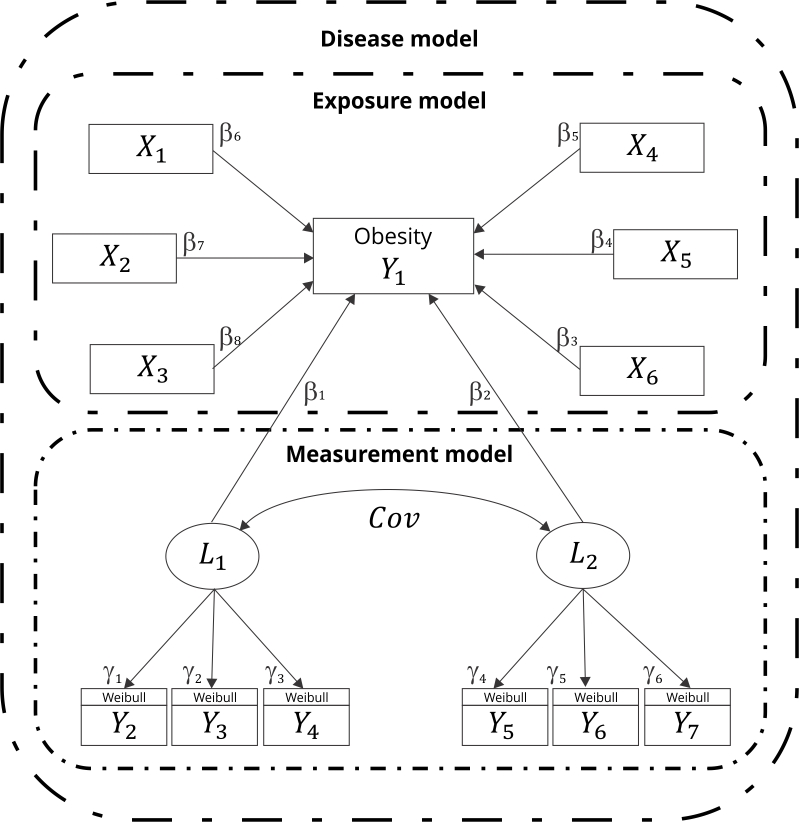
Disease theoretical model adopted for obesity occurrence in the adult population of the *Colombian National Survey of Nutritional Status*, 2015.

Note: *Y*_*1*_ is the outcome variable; *X*_*n*_ and *Y*_*n*_ are the covariables; *L*_*1*_ and *L*_*2*_ are the exogenous variables; and *γ*_*n*_ and *β*_*n*_ are the coefficients. The arrows indicate the linear effect of each variable on the latent variable. The curve connecting the outlined latent constructs reflects the covariance between these variables.

A GSEM was proposed, combining exposure and measurement models to define a disease model, as graphically illustrated in Figure 1. As shown, measurement models were proposed to estimate latent (unobserved, represented by ellipses) variables from their constituent indicators (measurable variables with non-Gaussian distribution, represented by rectangles). In turn, the exposure model estimates the direct effects of some covariates on the expected value of obesity occurrence. Finally, the disease model combines the two aforementioned models integrating both direct and indirect effects of the covariates and the latent variables (representing specific physical activity-related behaviors, for example) on the outcome.

The modified Poisson regression model with a robust variance estimator ^25^ ^,^ ^26^ that adequately estimates the prevalence ratios (PR) was selected in this study, in comparison with the logistic regression model. When the outcome is binary, the exponential coefficients estimated by Poisson regression are risk ratios instead of incidence-rate ratios. Then, *Y*_*1*_ was considered the outcome variable, representing the obesity occurrence (1/0, yes/no), in which *π*(*x*) = *Pr* (*Y*_*1*_ = *1*∣*x*).

Since *π*(*x*) is a positive function, the logarithm link function is a natural choice for modeling the expected value, that is: *log*(*π*(*x*)) = *α* + *L*_*1*_ *β*_*1*_ + *L*_*2*_ *β*_*2*_ + *β*_*2*_ *X*_*1*_ + ··· + *β*_*8*_ *X*_*6*_ . Figure 1 shows this expression, in which the set of endogenous variables contains computer and smartphone use, television viewing, playing video games, passive (car) transportation, cycling, and walking as modes of transport indicators. The new dimensions or latent variables were expressed as *L*_*1*_ and *L*_*2*_ (exogenous variables). The covariates sex, age, energy intake, socioeconomic score, and leisure physical activity were also included.

Goodness-of-fit tests were applied to identify the probability function with the best fit for each variable distribution. Kaiser-Meyer-Olkin (KMO) was used to estimate sample size adequacy and the sphericity test was used to assess the correlation between the variables. Based on the factorial analysis, two measurement models were estimated to identify the factorial loads and the adequacy of the variables of the physical activity dimensions that are hypothesized to be associated with obesity.

The structural model was estimated by maximum likelihood with 95% confidence intervals (95%CI). Considering the Akaike information criterion (AIC) and the Bayesian information criterion (BIC), the most parsimonious model, with significant path coefficients and theoretical significance, was selected. The expansion factors supplied by the database were used to adjust the statistical analyses. Stata, version 14.0 (https://www.stata.com), and RStudio, version 2022.02.3 (https://rstudio.com/), softwares were used.

Then, a score coefficient was estimated for each latent dimension identified, using the Bayesian empirical prediction method. This score represents the degree of adherence that each subject has to each latent variable. Therefore, the tertiles of the distribution of the scores define three categories: low, moderate, and high levels of adherence.

### Mapping

At the population level, the prevalence of obesity (% of people with a BMI greater than or equal to 30) was estimated in the 32 departments of Colombia and its capital, and its spatial distribution (in quartiles) was mapped. The QGIS software, version 3.26.3 (https://qgis.org/en/site/), was employed to create a map illustrating the adherence to the latent dimensions that showed a significant association with obesity.

### Ethical aspects

The Ethics Committee of Profamilia, under *Resolution n. 8,430/1993* of the Colombian Ministry of Health, approved the ENSIN, as it complied with the guidelines established in the *Declaration of Helsinki*. The database used in this work is in the public domain.

## Results

Table 1 describes the sample of 9,658 participants (56.2% females, mean age 38.5±13.1 years). Obesity prevalence was 19.1% (95%CI: 17.8; 20.4), with a greater prevalence in females (23.4%) compared to males (13.7%) (p < 0.001). About 75% of the subjects had completed high school and a third belonged to the lowest wealth quartile. Most participants (90.9%) reported no ethnic affiliation. When we carried out a descriptive analysis stratified by the occurrence of obesity, most people with obesity were females (68.6%), the age group with the highest prevalence was 30-49 years (49.9%), and the vast majority (74.2%) had a high school education. All the sociodemographic variables were significantly associated with having obesity (yes/no).

**Table 1:** Sociodemographic characteristics of the total number of participants (%). *Colombian National Survey of Nutritional Status*, 2015.

Characteristics	Obesity (%)	No obesity (%)	Total (%)	p-value *
	n = 1,949	n = 7,709	n = 9,658	
Sex				< 0.001
Male	31.4	46.8	43.8	
Female	68.6	53.2	56.2	
Total	19.1	80.9	100.0	
Age group (years)				< 0.001
18-29	18.7	34.2	31.2	
30-49	49.9	42.3	43.8	
50-64	31.3	23.5	25.0	
Ethnicity				< 0.001
Afro-descendant	9.0	6.6	7.1	
Indigenous	1.8	2.1	2.0	
Other	89.3	91.3	90.9	
Schooling level				
Primary	19.0	15.1	15.9	0.006
Secondary	74.2	75.5	75.3	
Tertiary	6.8	9.4	8.9	
Wealth index (quartiles)				< 0.001
Low (Q1)	27.6	30.0	29.5	
Middle-low (Q2)	25.1	23.4	23.7	
Middle-high (Q3)	26.2	23.9	24.4	
High (Q4)	21.1	22.8	22.4	

* Chi-square test.

In addition, we found that 50% of the sample spent 140 minutes/week traveling by passive means of transport (motorized vehicles) and about 70 minutes on foot. Regarding sedentary behaviors, watching television is the activity to which people dedicate the most time (median 420 minutes/week), followed by using a computer or smartphone (45 minutes/week). In a comparative analysis, people with obesity spend more time watching television (63 minutes/week) than people without obesity (p < 0.001), and this latter group spends more time using computers and smartphones (p < 0.001). We also found a significant difference in terms of weekly minutes dedicated to leisure physical activity (p = 0.001), with a higher maximum value in people without obesity.

To estimate the GSEM, goodness-of-fit tests first indicated that the Weibull probability function is adequate for the asymmetry observed in weekly time spent on transportation and use of screens. For the measurement model, Bartlett’s sphericity test (p < 0.001) confirmed that the variables are correlated, as the similarity matrix was not an identity matrix. The overall KMO test value was 0.526.

**Figure 2:**
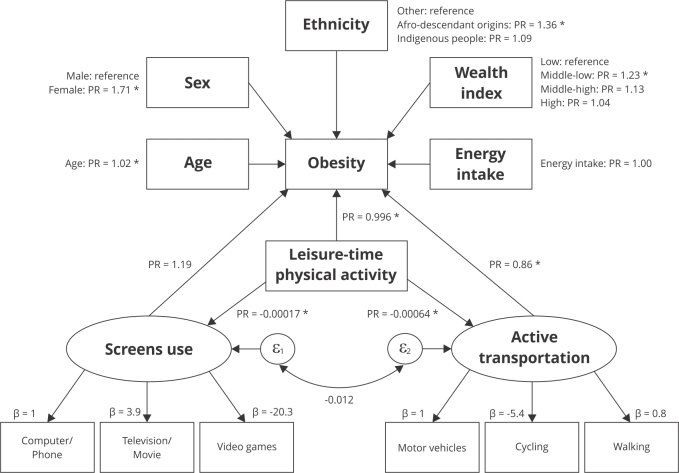
Estimation of direct and indirect effects among 9,658 participants of the *Colombian National Survey of Nutritional Status*, 2015.

Figure 2 illustrates the two latent variables, one called “Screens use” that represents the minutes per week in front of screens, including the use of computer and smartphone (as reference), television viewing (β = 3.9, p < 0.001), and use of video games (β = -20.3, p < 0.001) variables. The other, called “Active transportation”, is made up of the weekly minutes spent in transportation by motor vehicles (as reference), cycling (β = -5.4, p < 0.001), and walking (β = 0.8, p < 0.001).

β: estimated coefficients; ε_n_: latent error; PR: prevalence ratio. * p-value < 0.05.

As shown in Table 2, “Active transportation” was established as a protective factor for obesity (PR = 0.86, 95%CI: 0.7; 0.9), with the use of the bicycle being the most contributing indicator, detrimental to the use of passive transportation. The variable “Screens use” showed no significant effect on obesity.

**Table 2:** Estimation of direct and indirect effects of latent dimensions identified and selected covariates on obesity occurrence among 9,658 participants of the *Colombian National Survey of Nutritional Status*, 2015.

Effects	PR	95%CI	p-value
Direct			
Sex			
Male	Reference		
Female	1.71	1.55; 1.89	< 0.001
Age			
Year	1.02	1.01; 1.03	< 0.001
Ethnicity			
Other	Reference		
African	1.36	1.18; 1.58	< 0.001
Indigenous	1.09	0.89; 1.32	0.411
Wealth index (quartiles)			
Low (Q1)	Reference		
Middle-low (Q2)	1.23	1.10; 1.38	< 0.001
Middle-high (Q3)	1.13	1.00; 1.27	0.058
High (Q4)	1.04	0.90; 1.21	0.558
Intake energy			
kcal/day	1.00	0.99; 1.00	0.423
Leisure-time physical activity			
Minutes/week	0.9996	0.9994; 0.9999	< 0.001
Screens use			
Continuous index	1.19	0.69; 2.05	0.528
Active transportation			
Continuous index	0.86	0.75; 0.98	0.026
Indirect			
Leisure-time physical activity - Screens use	-0.00017	-0.00019; -0.000015	< 0.001
Leisure-time physical activity - Active transportation	-0.00064	-0.00068; -0.00059	< 0.001

95%CI: 95% confidence interval; PR: prevalence ratio.

Note: Poisson regression model (robust variance) adjusted for age, sex, schooling level, wealth index, physical activity, and total energy intake.

The exogenous variables were directly associated with obesity: age (PR = 1.02, 95%CI: 1.02; 1.03), sex (female: PR = 1.95, 95%CI: 1.8; 2.2), ethnicity (Afro-descendant: PR = 1.36, 95%CI: 1.18; 1.58, reference: no ethnic origin) and wealth index level (middle-low: PR = 1.26, 95%CI: 1.1; 1.4, reference: low). Conversely, the variable leisure-time physical activity was directly related to obesity as a protective factor (PR = 0.999, 95%CI: 0.998; 0.999) and indirectly through the dimension “Active transportation” (PR = -0.0006, 95%CI: -0.0068; -0.00059) and “Screens use” (PR = -0.0002, 95%CI: -0.00019; -0.00015).

**Figure 3:**
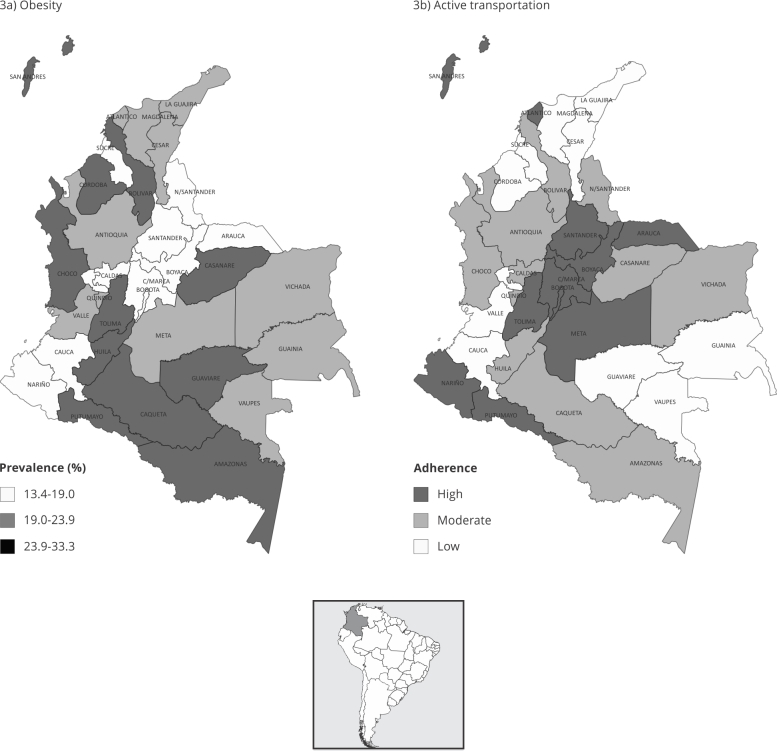
Spatial distribution of obesity burden (prevalence) and adherence level to the latent variable “Active transportation” (median score) in the adult population of the *Colombian National Survey of Nutritional Status*, 2015.

Figure 3a shows the spatial distribution of obesity prevalence in 32 departments and in Bogotá. The departments with higher prevalence are concentrated in the southwest of the country and in coastal departments. In total, 57% of all departments have a prevalence above the national average (19.1%).

Regarding adherence to the “Active transportation” dimension (Figure 3b), we found it to be mainly concentrated in the most densely populated and industrialized departments, which are located mainly in the center of the country. As already shown, this area presents low prevalence levels, below the national averages. This suggests possible coincidences of effects, in which the departments with greater “Active transportation” also have a lower burden of obesity prevalence.

## Discussion

The results of this research are pioneering in exploring the direct and indirect effects of weekly time spent in screens use and active transportation, as well as their association with obesity in adults in Colombia. Our latent dimensions, identified via a GSEM estimation, were named “Screens use” and “Active transportation”, according to their conformation indicators, in an attempt to define two interpretable dimensions. Both dimensions were related to the leisure time physical activity, but only the “Active transportation” dimension had a protective effect on obesity occurrence. In addition, it showed higher levels of adherence in the departments with better socioeconomic conditions and less prevalence of obesity.

### Active transportation

Although the literature has evaluated the impact of different forms of transportation on health, especially referring to injuries and pollution from motor vehicle emissions, the impact of means of transportation on BMI-related morbidity has not been explored in depth ^27^. Several systematic reviews indicate that evidence linking transport and obesity remains unclear ^13^ ^,^ ^18^ ^,^ ^19^; however, interventions promoting active transportation can provide benefits to prevent obesity. Cycling and walking in daily life are encouraged as part of strategies for obesity control and health promotion since active transportation shows health benefits, reduces air and noise pollution, and improves social and urban capital ^12^ ^,^ ^28^. In particular, our findings showed that the active transportation dimension, mainly represented by cycling as a means of transport, presents a protective effect against obesity. In line with this, some studies report that active commuting or transportation was associated with a lower risk of obesity and other non-communicable diseases among adults ^16^, as well as healthier body weight and composition in midlife ^17^. Similarly, the multi-country *Latin American Health and Nutrition Study* (ELANS, acronym in Spanish) found that time spent in active transportation was significantly associated with lower body mass index ^29^. These favorable effects may be related to the contribution of active transport behaviors to overall daily physical activity ^30^, increasing daily energy expenditure. However, the association between overall or leisure-time physical activity and other domains of physical activity such as active transportation remains not fully understood ^13^ ^,^ ^18^ ^,^ ^31^. As a contribution to this field, using a rigorous estimation procedure based on GSEM, we found a direct protective effect on the occurrence of obesity in people who practice physical activity in their leisure time, which in turn was related to active transportation. This fact is consistent with other studies, which state that associations with obesity in the expected direction have been found in people who use active means of transport and practice regular physical activity ^18^ ^,^ ^32^. Moreover, we highlight that our latent variable called active transportation was mainly characterized by cycling in contrast to motor vehicle commuting. Our results corroborate previous studies suggesting that car use may be associated with a higher risk of obesity ^10^ ^,^ ^33^. Taken together, these findings support the recommendation to choose or promote active transportation instead of passive transportation whenever possible.

### Screens use

The “Screens use” latent variable showed no significant effect on obesity. Although international organizations report that a sedentary lifestyle and physical inactivity are risk factors associated with all-cause mortality ^34^, many of which are obesity-related mortality causes, our results agree with reviews reporting inconclusive evidence in adults ^9^ ^,^ ^10^. However, the most consistent associations refer to screen time (mainly TV viewing) and markers of adiposity ^10^, similar to the results of our study, in which people with obesity reported spending more screen time watching television than people without obesity. To clarify the obesogenic effect of screen time, which appears to be stronger in children and adolescents than in adults, much more information is needed on the influence of potential confounder or mediator factors, including unhealthy dietary behaviors ^9^. Our study advances in this direction by presenting a theoretical model of obesity that incorporates, in the estimation process, selected adjustment variables (including total energy intake, leisure time physical activity, among others) and the potential covariation between the latent dimensions of screens use and active transportation. However, other relevant aspects not investigated in our study require further research. Some of the gaps identified in the literature in this field include the effects of frequent breaks from sitting, the role of unhealthy dietary patterns when using screens, and the consideration of different types of sedentary occupations and age groups ^9^ ^,^ ^10^.

### Social inequalities and spatial distribution

In our study, the spatial distribution of obesity also suggests that, in departments of the southwest and coastal areas, where the population is largely Afro-Colombian or has Indigenous origins, the prevalence reaches 20%. In contrast, in the other Mediterranean departments, which are more densely populated and economically developed, the prevalence of obesity was lower, even when including industrialized capital cities with transportation problems ^35^ ^,^ ^36^ ^,^ ^37^ ^,^ ^38^ ^,^ ^39^. In line with this, some studies have documented that ethnic minorities, especially Afro-descendants, are more likely to have a high prevalence of obesity ^40^. For the Latin America and the Caribbean region, as well as Colombia in particular, it has been highlighted that Indigenous and Afro-descendant peoples are ethnic-racial groups that are systematically exposed to multiple forms of material and social deprivation, trapped in a context of poverty and vulnerability ^41^ ^,^ ^42^. Moreover, the historical lack of statistical visibility of these communities has prevented adequate identification and recognition of the magnitude and various manifestations of the poverty they experience, including health impacts. The inequitable access to health, education, and employment services translates into substantial disparities in the quality of life and physical and mental health. In a cross-sectional study based on data from the 2019 *Colombian National Quality of Life Survey*, the authors concluded that ethnic-racial status is a structural component of inequity in access to health services and heightens the disadvantages of people with low socioeconomic status ^42^. This complex scenario could explain, in part, the unequal distribution of obesity across the Colombian territory.

In terms of public policies, Colombia presents a government plan of social development since 2014 that includes the use of bicycles. This policy promotes transportation in non-motorized forms, incorporation of road interconnection projects, construction of bikeways, and improvement of the regulatory framework for traffic on streets, highways, and bikeways. Although progress has been made in this field, given our results, public policies that encourage the use of active transportation must still be developed in remote areas where people of Indigenous and African origins mainly live (southwest and coastal areas).

### Limitations and strengths of the study

Recall bias may be one of the possible limitations in identifying physical activity dimensions, although the ENSIN survey excluded the population most susceptible to this type of bias, identified using a cognitive function test. In addition, naming latent dimensions may be subjective, limiting replication and comparability across studies. Moreover, we recognize the limitations of using BMI alone to classify nutritional status, as additional measures are important in the diagnosis of obesity. However, BMI was found to be the most useful measure of overweight and obesity at the population level ^43^. Despite these limitations, this work shows important strengths, including the use of a nationally representative probabilistic sample and standardized protocols. Moreover, the use of GSEM for data analysis allowed us to construct a theoretical model that can improve our understanding of the associations between the multiple factors involved in the development of obesity. For interpretation purposes, the Poisson regression model with a robust variance estimator, which was selected for this study, has been reported as one of the most consistent and efficient for estimating parameters of primary interest.

## Conclusion

Our results suggest that active transportation, mainly cycling (as opposed to non-active transportation), is a dimension of physical activity-related behaviors that may influence the burden of obesity, an association not found for screens use. Moreover, the spatial distribution of active transportation behaviors and obesity in Colombia seems to be shaped by social factors such as socioeconomic barriers, ethnic-racial inequalities, and limited economic development. These findings highlight the need for primary prevention interventions and public policies that promote social equity across regions, particularly in terms of access to infrastructure that encourages active transportation among the most socioeconomically disadvantaged populations. Finally, we emphasize that daily transportation choices are important factors to consider in public health recommendations, especially considering that cycling and walking to work have been highlighted as effective ways to incorporate regular physical activity into a sedentary lifestyle ^14^. This is particularly important given the context of declining levels of physical activity worldwide.

## References

[1] Phelps NH, Singleton RK, Zhou B, Heap RA, Mishra A, Bennett JE (2024). Worldwide trends in underweight and obesity from 1990 to 2022: a
pooled analysis of 3663 population-representative studies with 222 million
children, adolescents, and adults.. Lancet.

[2] Muñoz FL, Pou SA, Diaz MDP (2023). An empirically derived "prudent" dietary pattern is associated
with lower obesity occurrence modeling and mapping from a national nutrition
survey. Nutr Res.

[3] Popkin BM, Adair LS, Ng SW (2012). Global nutrition transition and the pandemic of obesity in
developing countries. Nutr Rev.

[4] González S, Sarmiento OL, Lozano Ó, Ramírez A, Grijalba C (2014). Physical activity levels among Colombian adults inequalities by
gender and socioeconomic status. Biomedica.

[5] World Health Organization Physical activity..

[6] Cuadri Fernández J, Tornero Quiñones I, Sierra Robles Á, Sáez Padilla JM (2018). Revisión sistemática sobre los estudios de intervención de
actividad física para el tratamiento de la obesidad. Retos.

[7] Jakicic J, Powell K, Campbell W, Dipietro L, Pate RR, Pescatello LS (2019). Physical activity and the prevention of weight gain in adults a
systematic review. Med Sci Sports Exerc.

[8] Marquez D, Aguiñaga S, Vásquez P, Conroy D, Erickson KI, Hillman C (2020). A systematic review of physical activity and quality of life and
well-being. Transl Behav Med.

[9] Rezende L, Rodrigues M, Rey JP, Matsudo V, Luiz O (2014). Sedentary behavior and health outcomes an overview of systematic
reviews. PLoS One.

[10] Biddle S, Bengoechea E, Pedisic Z, Bennie J, Vergeer I, Wiesner G (2017). Screen time, other sedentary behaviours, and obesity risk in
adults a review of reviews. Curr Obes Rep.

[11] Katzmarzyk PT, Powell KE, Jakicic JM, Troiano RP, Piercy K, Tennant B (2019). Sedentary behavior and health update from the 2018 Physical
Activity Guidelines Advisory Committee. Med Sci Sports Exerc.

[12] Winters M, Buehler R, Götschi T (2017). Policies to promote active travel evidence from reviews of the
literature. Curr Environ Health Rep.

[13] Saunders LE, Green JM, Petticrew MP, Steinbach R, Roberts H (2013). What are the health benefits of active travel A systematic review
of trials and cohort studies. PLoS One.

[14] Dinu M, Pagliai G, Macchi C, Sofi F (2019). Active commuting and multiple health outcomes a systematic review
and meta-analysis. Sports Med.

[15] Andersen LB (2016). Active commuting an easy and effective way to improve
health. Lancet Diabetes Endocrinol.

[16] Wu J, Li Q, Feng Y, Bhuyan SS, Tarimo CS, Zeng X (2021). Active commuting and the risk of obesity, hypertension and
diabetes a systematic review and meta-analysis of observational
studies. BMJ Glob Health.

[17] Flint E, Cummins S (2016). Active commuting and obesity in mid-life cross-sectional,
observational evidence from UK Biobank. Lancet Diabetes Endocrinol.

[18] Wanner M, Götschi T, Martin E, Kahlmeier S, Martin B (2012). Active transport, physical activity, and body weight in adults a
systematic review. Am J Prev Med.

[19] Brown V, Moodie M, Mantilla A, Veerman J, Carter R (2017). Active transport and obesity prevention a transportation sector
obesity impact scoping review and assessment for Melbourne,
Australia. Prev Med.

[20] Darbandi M, Najafi F, Pasdar Y, Mostafaei S, Rezaeian S (2020). Factors associated with overweight and obesity in adults using
structural equation model mediation effect of physical activity and dietary
pattern. Eat Weight Disord.

[21] Tarka P (2018). An overview of structural equation modeling its beginnings,
historical development, usefulness and controversies in the social
sciences. Qual Quant.

[22] Ministerio de Salud y Protección Social (2015). Encuesta Nacional de Situación Nutricional en Colombia - 2015.

[23] World Health Organization Obesity and overweight..

[24] Medina C, Barquera S, Janssen I (2013). Validity and reliability of the International Physical Activity
Questionnaire among adults in Mexico. Rev Panam Salud Pública.

[25] Cummings P (2009). Methods for estimating adjusted risk ratios. Stata J.

[26] Zou G (2004). A modified Poisson regression approach to prospective studies
with binary data. Am J Epidemiol.

[27] Nazelle A, Nieuwenhuijsen M, Antó J, Brauer M, Briggs D, Braun C (2011). Improving health through policies that promote active travel a
review of evidence to support integrated health impact
assessment. Environ Int.

[28] Filigrana P, Levy J, Gauthier J, Batterman S, Adar S (2022). Health benefits from cleaner vehicles and increased active
transportation in Seattle, Washington. J Expo Sci Environ Epidemiol.

[29] Habinger J, Chávez J, Matsudo S, Kovalskys I, Gómez G, Rigotti A (2020). Active transportation and obesity indicators in adults from Latin
America ELANS Multi-Country Study. Int J Environ Res Public Health.

[30] Celis-Morales CA, Lyall DM, Welsh P, Anderson J, Steell L, Guo Y (2017). Association between active commuting and incident cardiovascular
disease, cancer, and mortality prospective cohort study. BMJ.

[31] Menai M, Charreire H, Feuillet T, Salze P, Weber C, Enaux C (2015). Walking and cycling for commuting, leisure and errands relations
with individual characteristics and leisure-time physical activity in a
cross-sectional survey (the ACTI-Cités project). Int J Behav Nutr Phys Act.

[32] Smith L, Stubbs B, Hu L, Veronese N, Vancampfort D, Williams G (2019). Is active transport and leisure-time physical activity associated
with inflammatory markers in US adults A cross-sectional analyses from
NHANES. J Phys Act Health.

[33] McCormack GR, Virk JS (2014). Driving towards obesity a systematized literature review on the
association between motor vehicle travel time and distance and weight status
in adults. Prev Med.

[34] Lavie C, Ozemek C, Carbone S, Katzmarzyk PT, Blair SN (2019). Sedentary behavior, exercise, and cardiovascular
health. Circ Res.

[35] Prada E, Camargo D, Férmino R (2021). Participation and physical activity in recreovia of Bucaramanga,
Colombia. J Phys Act Health.

[36] Ospina J, López V, Botero V, Duque J (2020). A database to analyze cycling routes in Medellin,
Colombia. Data Brief.

[37] Ramírez R, Beltrán C, Correa J, Vivas A, Prieto D, Martínez J (2016). Factors associated with active commuting to school by bicycle
from Bogotá, Colombia The FUPRECOL study. Ital J Pediatr.

[38] Gómez LF, Mosquera J, Gómez OL, Moreno J, Pinzon JD, Jacoby E (2015). Social conditions and urban environment associated with
participation in the Ciclovia program among adults from Cali,
Colombia. Cad Saúde Pública.

[39] Arellana J, Alvarez V, Oviedo D, Guzman LA (2021). Walk this way pedestrian accessibility and equity in Barranquilla
and Soledad, Colombia. Research in Transportation Economics.

[40] Lee A, Cardel M, Feingold K, Chrousos G (2019). Social and environmental factors influencing
obesity.. Endotext.

[41] Comisión Económica para América Latina y el Caribe (2016). La matriz de la desigualdad social en América Latina.

[42] Viáfara CA, Palacios G, Banguera A (2021). Ethnic-racial inequity in health insurance in Colombia a
cross-sectional study. Rev Panam Salud Pública.

[43] Pou SA, Diaz MDP, Velázquez GA, Aballay LR (2021). Sociodemographic disparities and contextual factors in obesity
updated evidence from a National Survey of Risk Factors for Chronic
Diseases. Public Health Nutr.

